# “Cigarettes play the equalizer”: discrimination experiences and readiness to quit cigarette smoking among African Americans experiencing homelessness: a qualitative analysis

**DOI:** 10.1186/s13722-023-00432-8

**Published:** 2024-01-02

**Authors:** Alexandria Jones-Patten, Sanghyuk S. Shin, Adeline Nyamathi, Dawn Bounds

**Affiliations:** 1 Columbia University School of Nursing, Center for Research On People of Color, 560 W 168Th St, New York, NY 10032 USA; 2 Irvine School of Nursing Berk Hall, University of California, 802 West Peltason Drive, Irvine, CA 92617 USA; 3 Columbia University Irving Medical Center New York, New York, NY USA

**Keywords:** African Americans, Homelessness, Cigarette smoking, Discrimination

## Abstract

**Background:**

Approximately 70–80% of people experiencing homelessness in the United States use tobacco. Smoking cessation programs specifically for this population have been found to be less effective for African American participants. The purpose of this study was to explore discrimination experiences and their impact on smoking habits and readiness to quit cigarette smoking while experiencing homelessness.

**Methods:**

In the qualitative phase of this mixed methods study, five focus groups were conducted for African Americans residing in a homeless shelter in Skid Row, Los Angeles, CA. Using a semi-structured interview guide, we asked participants about discrimination experiences, how smoking habits were impacted by these experiences, and tools needed to successfully abstain from cigarette smoking. Qualitative descriptive content analysis was used to explore discrimination experiences and its association with readiness to quit cigarette smoking.

**Results:**

Of the 17 participants, 14 (82.4%) were male, and the average age was 46.8 years. Using a qualitative In Vivo coding method, three themes were revealed: “*Experiencing Discrimination while Black*”, “*The Psychosocial Fabric—Why Quitting Cigarette Smoking is a Challenge*”, and “*The Lesser of Two Evils—Choosing to Smoke over More Harmful Options.”* Participants discussed working in the blue-collar workforce while Black, identifying as a double minority, smoking to cope with stress, early exposure to cigarettes*,* smoking being a central part of one’s belonging to a group, and the legality of cigarette smoking.

**Discussion:**

Our findings show that African Americans experiencing homelessness (1) may experience discrimination in multiple settings, regardless of housing status, (2) could have grown up around cigarette smoking and remain surrounded by it while experiencing homelessness, and (3) may experience a calming effect with smoking, which slows some from reacting negatively to adverse situations.

**Conclusion:**

Barriers to successfully abstaining from smoking are multifactorial among African Americans experiencing homelessness and should be addressed individually. Future research should explore the cultural tailoring of interventions that support cessation efforts unique to minoritized populations to improve smoking cessation programs offered to this population.

## Background

More than 560,000 people experience homelessness in the United States (U.S.) on a given night; nearly 40% of them are African American (AA), [21]. Reasons for homelessness seem endless, but most often include poverty, lack of employment, incarceration, high housing costs and evictions, mental illness, and discrimination [6, 45]. Compared to housed individuals, people experiencing homelessness (PEH) have a shorter lifespan, largely because of the high prevalence of cigarette smoking, which is associated with heart disease, cancer, stroke, lung disease, and unintentional injury [16].

Approximately 70–80% of PEH in the U.S. use tobacco [33], while the prevalence of cigarette smoking by race/ethnicity among adult PEH remains unclear. Reasons for continued smoking among this population include socialization, which may stem from smoking in designated smoking areas within a shelter, and cigarette smoking being used as a coping mechanism for managing stress [1, 6, 40]. Smoking cessation programs specifically for PEH have been implemented; however, research in this area is sparse. The Power to Quit Study [18] is one of the largest smoking cessation intervention trials to date among PEH (n = 430), which assessed the effectiveness of motivational interviewing while participants received nicotine replacement therapy for eight weeks. At the 26 week follow up there were no statistically significant differences in 7-day abstinence from cigarette smoking between the intervention and control groups (9.3% vs. 5.6%, p = 0.15), [28]. Some potential reasons for the null study findings may include that the motivational interviewing intervention was not culturally tailored for the social context of homeless shelters, nor did it address a potential connection between cigarette use and discrimination.

Discrimination is differential treatment of the members of different ethnic, religious, national, or other groups or identities [4]. Research examining the relationship between cigarette smoking and discrimination, particularly while experiencing homelessness is limited, although there are several studies which examine this relationship among housed populations. Kendzor and colleagues [25] used an online survey to explore discrimination experiences and cigarette smoking among Whites, AAs and Latinos, and found that discrimination experiences were positively associated with cigarettes smoked per day, heaviness of smoking, self-rated addiction, and primary and secondary dependence. Using larger cohort data, evidence suggests that Blacks/African Americans who report any experiences of discrimination have greater odds of being a cigarette smoker [10, 38]. Similar results were found using data from the National Survey on American Life, which included Afro Caribbean (n = 1312) and African American (n = 3150) adults; those who reported experiencing everyday discrimination had higher odds of being a current cigarette smoker [36].

Studies among housed individuals suggest smoking cessation programs are less effective among AAs than their White counterparts. For example, Nollen et al.’s [27] study among 224 Black and 225 White smokers found smoking abstinence of 14.3% and 24.4% among Black and White people who smoke, respectively, at week 26 of a prospective intervention trial (odds ratio [OR] 0.51, 95% confidence interval [CI] 0.32–0.83, p = 0.007). Stevens et al.’s study (2016) among a cohort of 34,393 patients aged 18 and older in six diverse health care organizations found lower smoking abstinence among AAs vs. Whites who smoke (OR: 0.84, 95% CI 0.75–0.94, p = 0.002). It is suggested that barriers to smoking cessation, specifically among AAs experiencing homelessness (AA PEH) may include low self-efficacy [32], nicotine dependence, homelessness, and fatalism [42], as well as work, legal, and family problems [41].

Another potential barrier to smoking cessation specifically among AA PEH could be discrimination experiences. In the general AA population, perceived racial/ethnic discrimination, has been inversely associated with 7-day point prevalence abstinence among Black adults [23]. A smoking cessation intervention trial found lack of home ownership, lower income, greater neighborhood problems, and higher baseline cotinine to be factors which affected smoking abstinence of Blacks compared to White participants [27]. While experiences of discrimination among PEH have been infrequently studied, available research suggests that discrimination is more likely to be reported among AAs than Whites (AA: 48.11% vs. White: 14.76%, [29]),mean discrimination scores: AA: 33.17 (58.44), White: 17.68 (38.31), [46]). Discrimination has also been linked to the experience of being homeless [29, 39], as well as substance use and stressful life events among PEH [2].

Research exploring readiness to quit cigarette smoking (a measure of one’s readiness to engage in smoking cessation) particularly among PEH is also limited. While one-third of PEH who smoke report readiness to quit smoking within the next 6 months, nearly one-fifth are ready to quit within the next month [17]. Among PEH who smoke, greater readiness to quit cigarette smoking has been associated with higher subjective social status [17] and increased number of quit attempts [1]. To our knowledge, no studies have explored discrimination experiences and readiness to quit cigarette smoking among AA PEH. The purpose of this study was to identify whether PEH’s experiences of discrimination influence their cigarette smoking habits and their readiness to quit smoking.

### Conceptual framework

The Transtheoretical Framework (TTM), developed by Prochaska and colleagues [34], also called the Stages of Change Model, assumes that individuals move through the following stages of change (at times back and forth): precontemplation, contemplation, preparation, action, and maintenance, and termination. We chose this model for this study because it may best resemble the thought process involved in smoking cessation of this study population. That is, cigarette smokers may shift back and forth between various stages of this model while attempting to quit smoking. We believe these shifts may be at least partially explained by their environment (e.g. home, work) and social support systems. This framework has been used in studies exploring substance use among PEH and their readiness to quit [41], providing mixed results regarding participant readiness to quit cigarette smoking. We believe that as discrimination experiences occur more frequently, readiness to quit will decline.

## Methods

### Design

We used a qualitative descriptive study design with participants recruited from a larger, quantitative study (Jones-Patten et al., Manuscript submitted for publication). Briefly, the quantitative study used a cross-sectional survey design to explore the association between perceived discrimination (Everyday Discrimination Scale, [44] and readiness to quit cigarette smoking (Contemplation Ladder, [9] using a convenience sample of 100 participants from a large homeless shelter in Skid Row, Los Angeles, CA. Data from all 100 participants were collected between February and May, 2022. Current cigarette use, including heaviness of smoking (Heaviness of Smoking Index, [20]), and other substance use (TCU Drug Screen 5, [26]) were assessed. Sociodemographic data were also collected and included participants’ age, race, gender, and homeless and smoking histories.

Using a semi-structured interview guide (Table 1), we conducted focus groups of up to five participants per group to explore discrimination experiences, responses to those experiences, how they have impacted smoking habits, and what participants believed they would need to abstain from cigarette smoking.

**Table 1:** Focus Group Discussion Questions

1. Thinking back to a time that stands out in your mind when you felt discriminated against, what happened and how did you respond?
2. How have these feelings of discrimination affected your smoking habits?
3. Describe your first experience with smoking cigarettes. How old were you? What was happening at that time in your life?
4. Describe a time when you tried to quit smoking cigarettes. What stopped you from quitting? How did you feel about attempting to quit smoking?
5. What would need to happen in your life right now to quit cigarette smoking in the next 30 days? The next 6 months?

### Inclusion criteria

The quantitative phase of the study required participants were at least 18 years of age, self-identified as AA, self-reported being a current cigarette smoker, were experiencing homelessness for at least 30 days, and did not demonstrate cognitive impairment, based on a decision-making capacity tool completed prior to study enrollment. Participants were invited to participate in the qualitative study (focus groups) if they participated in the quantitative study and provided verbal consent to participate in audio-recorded focus group discussions. While all 100 participants from the quantitative study were initially considered eligible to participate in the focus groups, due to COVID-19-related restrictions only participants who still resided in the study’s host shelter at the time of the focus groups were eligible to participate.

### Sample and setting

We used a convenience sample for this study. Focus groups were conducted in a private room at a large shelter in Skid Row, Los Angeles, CA.

### Recruitment

Participants from the quantitative study who resided in the shelter were contacted by phone and provided the qualitative study information of the proposed study.

### Procedures

Participants engaged in audio-recorded, semi-structured focus groups in a private room. One to two focus groups were held every week for participants until data were saturated, and no new concepts were introduced. Participants created and wrote a pseudonym on a name tag to be worn during the interview. Study information sheets were read to participants prior to beginning recordings, and all questions were answered prior to obtaining consent to participate. Verbal consent was then collected before the initiation of the audio recording. Focus groups were facilitated by the lead author, with a second trained research team member present to assist with keeping participants focused on the questions asked and note taking.

After the sessions, audio recordings were uploaded to Microsoft Word, with transcription provided for each recording. The lead author listened to each recording while following along with the transcript created, using an abridged transcription method to edit transcripts when the dictation was incorrect. Transcription of content from focus groups was then uploaded to Dedoose Cross Platform application (Dedoose Version 9.0.54) for analysis within 1 week after each focus group concluded.

### Data analysis

We performed descriptive statistics for scores of everyday discrimination (range: 9–54) and readiness to quit smoking (range: 0–10) variables, and covariates including age, education, heaviness of smoking (range: 0–6), and TCU Drug Screen V scores (range: 0–11). Qualitative In Vivo coding [35] using Dedoose software was conducted to create codes from transcripts. Transcripts from interviews were read from start to finish, with some note-taking. Transcripts were read line-by-line to capture key themes. Using an inductive approach, codes were created from the transcripts to group into categories and then themes. The lead author initially coded the interviews independently, summarizing common themes. A second researcher then assessed the transcripts independently, summarizing recurrent content, and then collaboratively discussed coding similarities and differences with the lead author. The final author, who was present for all focus groups, assessed the transcripts and themes by the lead author and second researcher when the two could not reach an agreement on themes.

Trustworthiness of qualitative data entails establishing a rigor to the research, providing validation through establishing credibility, dependability, transferability, and confirmability [3, 19]. To ensure the trustworthiness of our data, several methods were employed. First, to ensure credibility of the focus group transcripts, focus groups were attended by the lead author and a second qualitative researcher, the final author. Additionally, two researchers independently coded the transcripts. To ensure dependability, the two researchers attending the focus group sessions debriefed about these sessions immediately following their conclusion, and subsequently met several times to discuss and justify the codes and themes created. Transferability was ensured by asking open-ended questions to focus group participants and note-taking of responses. Additionally, the purpose and methods of the study were provided to participants using a study information sheet. Confirmability was ensured by creating an audit trail of audio recordings of focus groups, documented meetings with the study’s team members, and creating and revising study timelines. This study received approval from University of California, Irvine’s Institutional Review Board.

### Findings

Sample characteristics of participants are included in Table 2. Of the 100 participants from phase I of the study, 17 participated in one of five focus groups held (Table 3). The size of the focus groups ranged from two to four participants each. The time in discussion across focus groups ranged from 35 to 60 min. Of the 17 participants, 14 (82.4%) were male, and the average focus group participant age was 45.11 years. Five participants (29.4%) did not complete high school, six (35.3%) completed 12th grade, four (23.5%) completed trade school or took college courses, and two (11.8%) obtained a college degree. The mean discrimination scores of this sample was 31.59 (10.39). Mean contemplation ladder scores of focus group participants was 5.6; according to the contemplation ladder these scores indicate participants think they should quit smoking, but are not quite ready.

**Table 2:** Participant Demographics

Variable	Participants (n = 17)
Male sex, n (%)	14 (82.4%)
Age (mean, SD)	45.11 years; SD 14.10
Education	
Less than High School, n (%)	5 (29.4%)
High School, n (%)	6 (35.3%)
Trade School, Some College, n (%)	4 (23.5%)
College Completion, n (%)	2 (11.8%)
Days homeless (mean, SD)	276 days (~ 9.2 months); SD 435.56
Everyday Discrimination Scores (mean, SD)	31.59; SD: 10.39
Contemplation Ladder Scores (Readiness to Quit Smoking), (mean, SD)	5.6; SD: 3.14
Heaviness of Smoking Scores (mean, SD)	2.35; SD: 1.69
TCU Drug Screen Scores (mean, SD)	2.17; SD 3.00

**Table 3:** Focus Group Participant Pseudonyms and Age

	Group Number	Age	Gender
*Pseudonym*	–	–	–
Buddy Love	1	34	Male
Rosta	1	53	Male
Jason	1	59	Male
Ray	1	57	Male
Alfred	2	27	Male
Saadiq	2	31	Male
Big Boi	2	60	Male
Phil	3	30	Male
Rick	3	30	Male
James I	4	45	Male
James L	4	64	Male
Mr. “M”	4	63	Male
John	4	51	Male
Box	5	31	Female
Mario	5	34	Male
Jackie	5	63	Female
Bebe	5	35	Female

Three major themes were identified. The first was *Experiencing Discrimination while Black.* Participants described experiences of discrimination while seeking healthcare and while growing up. Subthemes included: *Working in the blue-collar workforce while Black, Identifying as a Double Minority: being Black and..,* and *Smoking More to Cope with Discrimination*. The second theme identified was *The Psychosocial Fabric—Why Quitting Cigarette Smoking is a Challenge* in which cigarettes were perceived as a way to belong in one’s community. Subthemes included *Early Exposure to Cigarettes, Smoking Surrounds Me* and *Cigarettes are Legal.* Finally, the third theme, *The Lesser of Two Evils—Choosing to Smoke Tobacco over more Harmful Options*, included participants' experiences with quitting other substances and how smoking may stop them from aggressively responding to a stressor. Subthemes included *I Quit All the Other Stuff* and *Smoking instead of Reacting/Fighting.*

### Experiencing discrimination while Black

When asked about discriminatory experiences, most responses centered around race. Subthemes include *Working in the blue-collar workforce while Black, Identifying as a Double Minority: being Black and..,* and *Smoking More to Cope with Discrimination*.

A participant who identified as “Box” describes not feeling accepted while walking down the street, “We walking down the street and… Why are ya’ll grabbing your purse? Just because we’re Black?”.

Ray described the first time being called a racial slur, shortly after returning to live with his mother following time he spent in the foster care system.

And it was little kids out there playing. And… we went out there to play... So, as we got through playing with them, this white lady comes out and she’s hollering to the kids to come here and… Then I heard the word “n*gga.”

Other notable experiences of discrimination occurred in the workplace, with participants describing their experience working in blue collar America.

### Working in the blue-collar workforce while Black

Several participants shared discriminatory experiences occurring in the workplace. One example of workplace discrimination included feeling less supported by managers of a different race. Big Boi described his experience working in a hospital in Texas,

I had this boss... He grew up in an environment where… Blacks didn't have nothing. So I was under the impression that… if I did something and then another person from another race did something it was OK for him to do that. But it wasn't OK for me to do it.

Another example of workplace discrimination offered was in the context of providing service to customers who commented on one’s race. Saadiq shared,

I was working at Home Depot at the time and there were multiple instances where customers came in and… did not agree with certain things. And just because of all the tension that was going on, would blurt out racial slurs… I was called the “n” word…[and told] “You need to go back to Africa.”

Still, several participants described the intersectionality of experiencing discrimination while identifying as a double minority.

### Identifying as a double minority: “being Black and…”

One female participant shared her story of a man stepping in front of her while in line at a local store.

I was in a Sears store in line… and a Hispanic… or Latino man came and just jumped in front of me… I didn't say nothing at first. I… thought he was like, ‘I'm getting in line,’ and didn't notice that I was there. He never moved and I was like “Excuse me, but you jumped in front of me… He said ‘I didn't see you.’

Rosta described growing up with a medical condition that he was unable to hide and receiving unwanted attention. “As a youth I was born with cerebral palsy, so I wore braces and cables. So that brought a lot of discriminatory attention my way from family, from friends, and from enemies of all colors.” Participants described how these experiences sometimes led to smoking a cigarette.

### Smoking more to cope with discrimination

In their responses to discrimination, participants described how discrimination impacted their smoking habits. James L. discussed using cigarette smoking to manage frustration following discrimination experiences. “It makes me very angry. It just makes me want to just continue smoking.” Mario provided a similar response to dealing with discrimination felt from others. “That makes me wanna smoke more.” The responses to discrimination often involved reflecting on cigarette smoking to regain control of their emotions. The socialization component attached to cigarette smoking was also discussed.

### The psychosocial fabric—why quitting cigarette smoking is a challenge

Cigarette smoking was described most frequently in social settings. Subthemes include *Early Exposure to Cigarettes, Smoking Surrounds Me* and* Cigarettes are Legal.*

Participants described their first experiences with cigarette smoking and the psychological troubles experienced trying to break away from cigarette smoking. Mario reflects, “I feel like… the more good things I do, the more I want to reward myself, and sometimes that cigarette is the most rewarding thing.”

Jackie recalls, “I stopped one time for 10 years because I started back going to church and I got healed. But then one time I tried to quit on my own. As soon as I got upset, I went right back.” Several participants discussed exposure to cigarettes at a young age either in the home or at school, and the legality of cigarettes.

### Early exposure to cigarettes

Participants described growing up around cigarettes in the home and starting to smoke as early as eight years old. Participants saw their siblings, parents, and caretakers smoking cigarettes growing up. Buddy Love reflected on being exposed to a tobacco farm a family member owned. “I just started smoking and he gave me what I want… I’d sell it in school and everything like that to the teachers… I was in middle school.” Similarly, James L. described access to cigarettes from family members while growing up. “I've been smoking ever since I was eight years old. And the only reason why I started smoking 'cause… My oldest brother hooked me on to smoking.” Other participants described the involvement of smoking in other facets of life, including at school and work.

### Smoking surrounds me

Many participants shared their experience with cigarette smoking with peers. Big Boi explained his first experience gave him a high that he still seeks from cigarettes today.

I think I might have been about 12 years old… I just walked in to use the restroom. It was like 10-12 guys in there… And a guy said, “Hey man, you want to hit this? I hit that cigarette, I was high as a kite. And then after that… I've been wanting a cigarette ever since then.

Bebe agreed. “My mom smoked my entire life, and my sister. I just did what everybody else did.” Rick’s reply was similar, stating hanging out with coworkers encouraged him to smoke, “A lot of my coworkers would smoke and I think I would pick up a cigarette… mostly, because they did. And it would feel awkward if I didn't, you know?” Participants discussed cigarette smoking being socially acceptable, but also being a legal way to relax.

### Cigarettes are legal

Participants reflected on the acceptability of cigarette smoking. Some were encouraged to take smoke breaks at work to calm themselves down following a negative experience. Saadiq noted, “The managers were like go take a break, go smoke, get you something, you know… and calm down.” Alfred discussed how cigarette smoking is more accepted in social settings than other substances,

I smoke more weed than I do cigarettes. But from an environment where I can't smoke weed, I gotta run way down the street, go smoke some weed and I gotta go around the corner two blocks... I'ma just smoke the cigarette.

The responses about the legality of cigarette smoking also led to discussion about why other substances are used less frequently, if at all.

### The lesser of two evils-choosing to smoke tobacco over more harmful options

Participants also discussed choosing cigarettes because they appear to be less harmful than engaging in other substance use or exhibiting physical aggression. Subthemes include *I Quit All the Other Stuff* and *Smoking instead of Reacting/Fighting.*

James L. reflects on smoking other substances in the past, but feels unable to quit cigarette smoking. “I quit drugs and alcohol, and all that stuff, but I just can't quit these cigarettes.” Buddy Love describes cigarettes as meditation, calming the urge to be aggressive,

I started smoking because I thought it was like a meditation. I thought it was going to ease everything... Sometime when I get in a fight or something I get real mad... I say man, I have to smoke a cigarette or I might have to lay this brother down.

More participants expressed quitting other substances thought to be more harmful and further discussed cigarette smoking to minimize the urge to become aggressive with someone.

### I quit all the other stuff

Other substances were thought to be more harmful because they caused participants to feel as though they were losing control. Several participants described a hierarchy when it came to substance use, and cigarettes were one of the last substances to quit.

Rosta shares his urges for other substances he believes is kept at bay by cigarettes.

Ultimately, as to right now, I'm down to cigarettes, is what I can say and, I know that you know they're not good… But because of those tendencies, those urges that I used to once respond to, the want or drive to smoke something is definitely still there, so I'm just trying to keep it contained to cigarettes.

Ray agreed, stating that he’s stopped using other substances. “And as I've gotten older, I notice it's kind of hard to stop the cigarettes. I stopped the drugs but the cigarettes was a little bit… harder, so I'm down… lower now.” Participants also shared how cigarettes stopped them from making decisions which may have dire consequences.

### Smoking instead of reacting/fighting

Participants reflected on their belief that cigarettes calmed urges to physically fight with someone, and reflected on potential consequences of reacting negatively or fighting with someone. Alfred describes smoking to avoid harmful physical acts toward others, which could land him in prison.

Cigarettes pretty much play the equalizer. Cigarettes give me a second to put everything into perspective… When I smoke a cigarette, I got 5 minutes of time to choose between my children and a bunch of dudes for the rest of my life… Cigarettes play a big part in not just calming down but… in the pros and cons and the… tomorrow.

To add, Box describes her thoughts on the potential consequences of physically harming someone else and how that may impact her currently living situation.

If I hit you, who will get in trouble, me or you? I'm the person that hit you first, right? That mean I'm going to jail... If I hit you, I’m looking at getting kicked out of here, and don't have nowhere to go, and I have to start back at square one.

## Discussion

Our study examined how discrimination experiences may impact readiness to quit cigarette smoking among African American (AA) people experiencing homelessness (PEH). The study findings revealed that AAs experience discrimination in multiple settings, regardless of housing status. For some, smoking cigarettes serves as an important coping response to manage stress. Furthermore, there is a psychosocial fabric around cigarette smoking, wherein cigarettes are introduced early in life and in social settings such as the home, work, or school environments, and participants feel urges to continue smoking or start again after initiating smoking cessation. To our knowledge, few studies have investigated readiness to quit cigarette smoking among AA PEH. Mean Contemplation Ladder (readiness to quit) scores for this study (5.6; SD: 3.2) suggests participants think they should quit, but are not quite ready, which is consistent with other literature [6].

### Discrimination experiences and minority status

Participants reported stressful and perceived discriminatory experiences in various settings (work, home, walking down the street); these experiences were described as catalysts for continued smoking. Wrighting and colleagues [46] surveyed PEH living in Oklahoma City, and found that of 115 Black participants sampled, Black adults experiencing homeless believed discrimination experiences were related to race (n = 30; 32.97%) followed by homeless (n = 22; 24.18%) and ethnicity (n = 11; 12.09%). Our study participants discuss identifying as a double minority, but do not regularly mention homelessness in this discussion. One reason for this could be that study participants knew each other and the experience of homelessness itself was assumed/unspoken because it was a known commonality among participants. Another reason may be that homelessness itself may not exacerbate discrimination as much or often as race/ethnicity. A study examining the relationship between race and the experience of homelessness among a predominately Black (80%) older adult sample found that (1) participants experienced overt racial discrimination in early life and (2) structural racism precipitated and perpetuated adult homelessness [31]. The experience itself of identifying as AA or Black may supersede other minority categories, including homelessness.

### Dependence on cigarettes

Several participants discussed cigarettes providing a calming effect for them, particularly in cases where they had physical responses to discrimination or another stressor. Cigarette smoking may provide some relief from physical signs of stress resulting from higher efforts to cope. Literature has suggested that the calming effect cigarette smokers describe may be the action of nicotine in ending withdrawal symptoms [37]. That is, as a smoker becomes addicted to nicotine they crave the relaxation which comes from smoking a cigarette, but this calming effect is actually nicotine working to keep withdrawal symptoms at bay. The use of cigarettes as a coping strategy may be an additional barrier to successful progression through the final Stages of Change model to achieve smoking abstinence. The calming effect participants described may be enhanced by their long-term relationship with cigarette smoking. The repeated experiences of discrimination may augment physical symptoms of stress, leading to the need or desire to smoke a cigarette to calm these symptoms. Future research should analyze physical symptoms associated with discrimination and cigarette use among racial/ethnic minorities.

Participants described cigarette use, at times, as a way to avoid physical altercations with others that could have consequences for their housing status. Our sample may be describing a limited amount of adaptive coping strategies, in which they turn to cigarette use in hopes it will settle uncomfortable feelings following a discriminatory or stressful experience. People experiencing homelessness may need to rely on personal attitudes and optimism as a source of coping and resilience [31], which may be thought to be more attainable when smoking cigarettes. Interventions targeting the use of resilience and other adaptive coping strategies may strengthen smoking cessation programs for AA PEH. As an example, a race-based, stress reduction intervention has been found to enhance the use of adaptive coping strategies, including emotional validation, and empowerment in response to racism and discrimination among a sample of veterans of color [12]. If such strategies were included in smoking cessation programs with a high prevalence of AA PEH, the gap in achieving smoking abstinence between AAs and Whites could potentially narrow.

### Psychosocial fabric to smoking

Participants described starting to smoke cigarettes at a young age and being exposed to cigarettes both in the home and at school while growing up, and smoking during work breaks with coworkers. Further, participants discussed the legality of cigarettes. While recreational and medicinal use of cannabis is legal in California, beyond cigarettes, other substance use is prohibited in many shelters, while designated cigarette smoking areas within some shelters remain. Cannabis use may also have influenced cigarette use among our participant sample. A nationally representative sample of adult cigarette and cannabis users from four different countries found that smokers who maintained or increased their consumption of cannabis had significantly lower odds of quitting cigarette smoking [15]. Among a sample of U.S. adults, cannabis use among never-smokers was associated with an increase likelihood of engaging in cigarette smoking and in smoking relapse [43]. This study did not collect data on cannabis use; however, future intervention programs around smoking cessation should examine cannabis and other substance use to analyze potential links between other substance use, smoking cessation and discrimination.

Regarding cigarette use, it is possible that our participants smoke cigarettes more often in social settings than alone in an effort to connect with others. In a study which sampled 626 men and women experiencing homelessness who were smokers, findings revealed that men who were actively smoking tobacco endorsed craving cigarettes and being around other cigarette smokers as the main barriers to quitting cigarette smoking, while women endorsed stress/mood swings, and coping with stress as the main barriers [11]. Indeed, designated cigarette smoking areas in one’s home environment coupled with an individual’s own ability to manage stress may endorse continued cigarette smoking. This can impede one’s chances of moving forward in the Stages of Change model and achieving smoking abstinence. While some shelters provide programs to mitigate substance use, they may inadvertently promote ongoing cigarette smoking by designating a space for this activity, especially in the social context. Additionally, smoking cessation programs may focus more on the individual, without also giving credit to the environment in which they live as a driving force to continue smoking. Smoking cessation programs could benefit from collaborating with shelters, especially those with designated smoking areas, to identify ways to limit socialization in designated smoking areas while promoting other opportunities to socialize within the shelter.

## Limitations

While this research adds to the literature on readiness to quit smoking among AA PEH, this study is not without limitations. First, our sample size is small. And with only three of the 17 participants being female, our results are primarily based on the perspectives of men. Second, we used an all-AA sample within a specific region of the United States, making it likely our results are not generalizable to other PEH across the U.S. Additionally, to remain consistent with each focus group discussion, we limited each discussion to 60 min. Two groups only covered the first three of five questions of the interview guide within the allotted 60 min. Despite these limitations, this research adds to the literature on barriers to smoking cessation among a vulnerable population. We identified several barriers to readiness to quit smoking among homeless adults, with potential interventions which should be considered when implementing smoking cessation programs among this population.

## Conclusion

The purpose of this study was to add to the literature on the complexities of abstaining from cigarette smoking among AA PEH. Barriers to successfully abstaining from smoking likely are multifactorial and may compete with both the greater need to cope with everyday life, and the socialization component that surrounds cigarette use within shelters. Future research should further investigate these barriers stemming from cigarette smoking while socializing. Smoking cessation programs designed for this population should include support for participants’ who smoke to feel welcomed and embraced in their community, and include developing interventions that support combatting discrimination, promote resilience, and enhance smoking cessation efforts for those who may be engaged in other substance use.

## Data Availability

The datasets generated and analyzed during the current study are not publicly available due the collection of personal data, but are available from the corresponding author on reasonable request.
